# Effect of Single-dose Azithromycin on Pneumococcal Carriage and Resistance: A Randomized Controlled Trial

**DOI:** 10.1097/INF.0000000000003585

**Published:** 2022-05-23

**Authors:** Boubacar Coulibaly, Dramane Kiemde, Guillaume Zonou, Ali Sié, Clarisse Dah, Mamadou Bountogo, Jessica Brogdon, Huiyu Hu, Elodie Lebas, Travis C. Porco, Thuy Doan, Thomas M. Lietman, Catherine E. Oldenburg

**Affiliations:** From the * Centre de Recherche en Santé de Nouna, Nouna, Burkina Faso; † Francis I Proctor Foundation, University of California, San Francisco, CA; ‡ Department of Ophthalmology, University of California, San Francisco, CA; § Department of Epidemiology & Biostatistics, University of California, San Francisco, CA.

**Keywords:** azithromycin, antimicrobial resistance, pneumococcus

## Abstract

We evaluated antibiotic resistance selection in *Streptococcus pneumoniae* isolates from children participating in an individually randomized trial of single-dose azithromycin versus placebo. After 14 days, the prevalence of resistance to erythromycin, oxacillin, and clindamycin was elevated in the azithromycin versus placebo group. There was no difference at 6 months.

Biannual mass azithromycin distribution has been shown to reduce all-cause childhood mortality in some high-mortality settings in sub-Saharan Africa.^1^ Any implementation program of azithromycin for childhood survival must include monitoring of selection for antibiotic resistance. Surveillance of antimicrobial resistance following mass azithromycin distribution for trachoma control has generally shown an increase in selection for macrolide resistance, with declines in the prevalence of resistance following cessation of antibiotic distribution.^2^ An increase in genetic macrolide resistance determinants has been observed in communities receiving biannual mass azithromycin distribution to children with 1–59 months of age compared with placebo.^3^ Individual-level antibiotic treatment likely also affects selection for resistance. We evaluated phenotypic resistance in *S. pneumoniae* isolates from children participating in an individually randomized trial of single-dose azithromycin versus placebo over a 6-month period.

## MATERIALS AND METHODS

### Study Setting

This study was conducted in Nouna Town, Burkina Faso. All children 3–59 months of age are eligible for seasonal malaria chemoprevention (SMC) with sulfadoxine-pyrimethamine monthly from June through October. Children were enrolled in November 2019, at the end of the malaria season and after all SMC distributions were complete. The final follow-up visit was in June 2020, and SMC was not distributed during the study period. Antibiotic use is relatively common in the study area, with children <5 years of age receiving approximately 2 antibiotic courses per year, most of which are amoxicillin.^4^

### Trial Methods

Complete methods for the trial have been previously described (clinicaltrials.gov NCT03676751).^5^ Children were eligible if they were between 8 days and 59 months of age at enrollment and were residents of Nouna Town, were able to feed orally, and had no known allergies to macrolides. Participants were randomized in a 1:1 fashion to a single oral 20 mg/kg dose of azithromycin or equivalent volume of matching placebo (Pfizer, Inc, New York, NY). All investigators, outcome assessors, and laboratory personnel were masked to the randomized treatment assignment. Nasopharyngeal swabs were collected at baseline before randomization and treatment, and 14 days and 6 months from enrollment. Swabs were placed in a cryotube with skim milk-tryptone-glucose-glycerin media on ice in the field. Samples were transported to the Centre de Recherche en Santé de Nouna laboratory and stored at –80°C until processing.

The Institutional Review Board at the University of California, San Francisco, the Comité d’Ethique pour la Recherche en Santé in Ouagadougou, Burkina Faso, and the Comite Institutionnel d’Ethique at the Centre de Recherche en Santé de Nouna in Nouna, Burkina Faso. Written informed consent was obtained from at least 1 guardian of each enrolled child.

### Laboratory Methods

A sample (0.1 mL) of skim milk-tryptone-glucose-glycerin from the cryotube containing the nasopharyngeal swab was plated and streaked on blood agar plates (Selective Strep Agar Base, CRITERION Dehydrated Culture Media, Hardy Diagnostics, Santa Monica, CA). *S. pneumoniae* was identified on selective media after incubation at 35°C in 5% CO_2_ by Optochin disk testing (Fisher Scientific, Hanover Park, IL). Antibiotic susceptibility testing was performed using Kirby-Bauer disc diffusion and included testing for erythromycin (15 μg), oxacillin (1 μg), tetracycline (30 μg), clindamycin (2 μg), and trimethoprim-sulfamethoxazole (23.75 μg/1.25 μg). Susceptibility was determined according to the Clinical and Laboratory Standard Institute zone diameter interpretive standards for *S. pneumoniae*.^6^ We considered intermediate and resistant isolates to be nonsusceptible. Isolates that were resistant to both erythromycin and clindamycin were considered to be because of the macrolide resistance mutation *ermB* and those resistant to erythromycin but not clindamycin were considered to be because of *mefA*.^7^

### Statistical Methods

We compared the proportion of isolates with pneumococcal carriage and, for isolates which grew pneumococcus, resistant to each antibiotic at each follow-up time point separately. We calculated binomial 95% confidence intervals (CI) for the prevalence of carriage and resistance to each antibiotic at each time point. An unadjusted logistic regression model with a single term for randomized treatment assignment was used to calculate odds ratios (OR) of carriage and resistance in children randomized to azithromycin versus placebo. A false discovery rate (FDR) correction was used to adjust *P* values for multiple comparisons. All analyses were conducted in R (The R Foundation for Statistical Computing, Vienna, Austria).

## RESULTS

**FIGURE 1.**
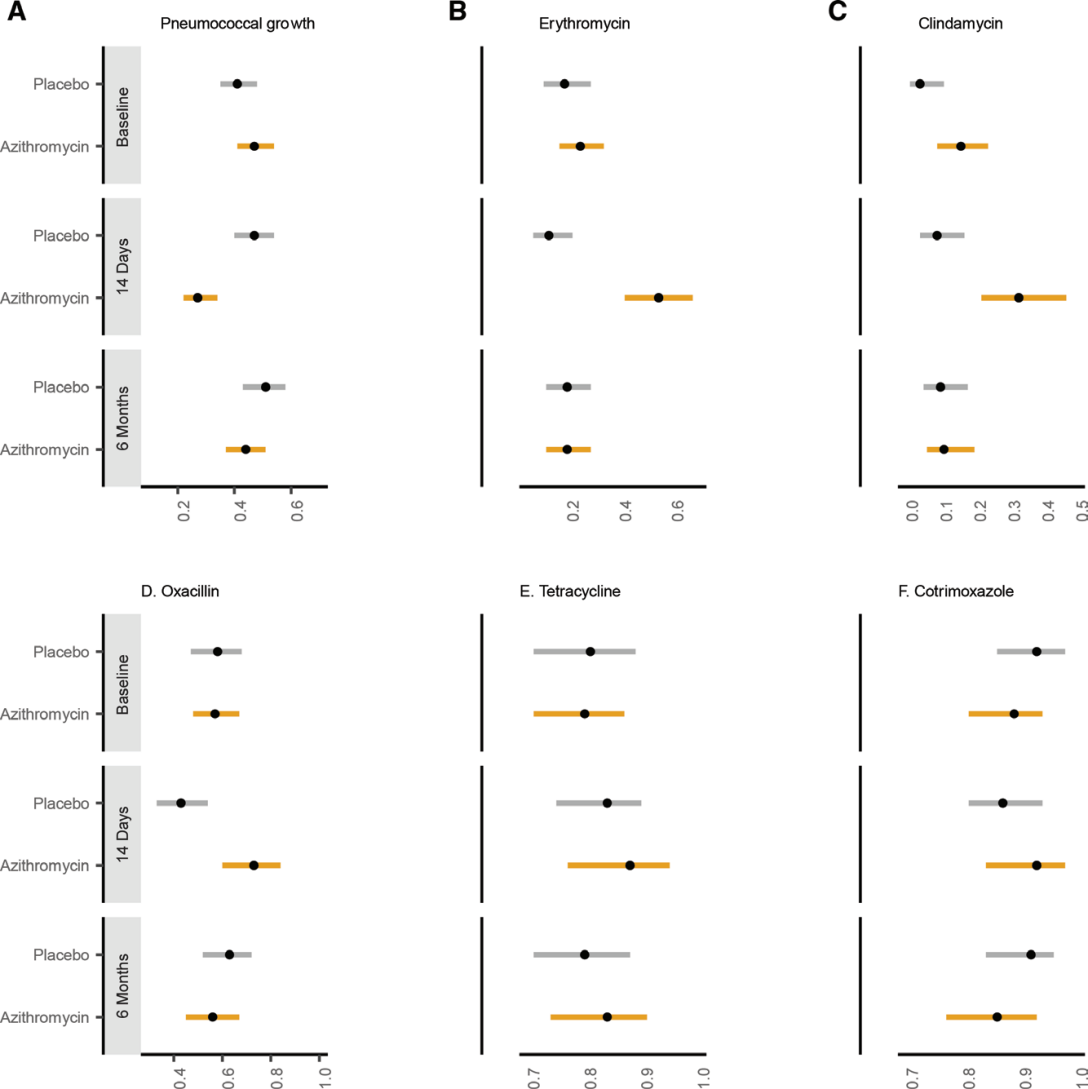
Prevalence of pneumococcal carriage (A), erythromycin (B), clindamycin (C), oxacillin (D), tetracycline (E), and trimethoprim-sulfamethoxazole (F) resistance by arm and study time point. Points indicate proportion of samples from which pneumococcus was isolated (for pneumococcal growth) or proportion of positive isolates that were resistant to the antibiotic. Bars indicate binomial 95% confidence intervals. Results from the placebo group are in gray and results from the azithromycin group are yellow.

Of 450 children enrolled in the parent trial, 230 were randomized to azithromycin and 220 to placebo (see Figure, Supplemental Digital Content 1, http://links.lww.com/INF/E740). At baseline, prevalence of pneumococcal carriage was 44% (azithromycin: 47%, placebo: 41%; see Table [Supplemental Digital Content 2, http://links.lww.com/INF/E740]). Among positive isolates, prevalence of resistance was similar between arms for all antibiotic classes at baseline (Fig. 1). Baseline characteristics were similar for children who were and were not lost to follow-up (see Table, Supplemental Digital Content 3, http://links.lww.com/INF/E740).

At 14 days after treatment, pneumococcal carriage was significantly lower in children receiving azithromycin compared with placebo: (azithromycin: 27%, placebo: 47%, OR = 0.43, 95% CI: 0.28–0.64, FDR-adjusted *P* < 0.001). Of the positive isolates, 55% in the azithromycin arm were resistant to erythromycin compared with 13% in the placebo arm (OR = 7.90, 95% CI: 3.72–17.65, FDR-adjusted *P* < 0.001). Oxacillin and clindamycin resistance were more common in the azithromycin group compared with placebo (oxacillin: azithromycin 73%, placebo 43%, OR = 3.60, 95% CI: 1.82–7.40, FDR-adjusted *P* < 0.001; clindamycin: azithromycin 33%, placebo 9%, OR = 4.89, 95% CI: 2.10–12.19, FDR-adjusted *P* < 0.001). Most (83%) isolates that were resistant to erythromycin were also resistant to oxacillin. ermB and mefA-induced macrolide resistance was more common in azithromycin compared with placebo-treated children (ermB: azithromycin 30%, placebo 8%, OR = 4.77, 95% CI: 1.98–12.45, FDR-adjusted *P* = 0.001; mefA: azithromycin 25%, placebo 5%, OR = 6.13, 95% CI: 2.22–19.83, FDR-adjusted *P* = 0.001). Tetracycline and trimethoprim-sulfamethoxazole resistance were similar between arms at 14 days (Fig. 1).

At 6 months after treatment, there was no significant difference in pneumococcal carriage between children receiving azithromycin compared with placebo (azithromycin: 44%, placebo: 51%, OR 0.75, 95% CI: 0.50–1.11, FDR-adjusted *P* = 0.92). Resistance prevalence was similar between arms at 6 months (Fig. 1; see Table, Supplemental Digital Content 4, http://links.lww.com/INF/E740).

## DISCUSSION

A single oral dose of azithromycin led to a short-term increase in phenotypic resistance to erythromycin in *S. pneumoniae*, with no evidence of long-term differences 6 months after treatment. The prevalence of erythromycin resistance returned to approximately baseline levels by 6 months after treatment, suggesting that azithromycin selected for macrolide resistance in the short term but did not result in persistent resistance selection. Studies of macrolide resistance in pneumococcus following mass azithromycin distribution for trachoma have found that community-level azithromycin treatment leads to short-term selection for macrolide resistance but that the prevalence of resistance declines over time following removal of selection pressure.^2^ The results from the present study suggest that a similar phenomenon occurs at the individual level, which is in line with previous observational studies.^8^

Oxacillin resistance was higher in children receiving azithromycin compared with placebo at 14 days. Previous studies have suggested an increase in genetic β-lactam resistance determinants in children living in communities receiving biannual mass azithromycin distribution compared with placebo after multiple rounds of treatment, although there was no difference at 60 months.^3,9^ Coselection for resistance to multiple antibiotic classes is possible if bacteria harbor resistance mutations to multiple antibiotic classes. Coresistance to both macrolides and β-lactams has been previously reported and appears to explain this finding in the present study as most erythromycin-resistant isolates were also resistant to oxacillin.^10^

This study has several limitations. The study was conducted in an area with relatively high use of amoxicillin and to a lesser extent cotrimoxazole, and less commonly macrolide usage. These results may not be generalizable to other settings if background antibiotic use affects selection for resistance differentially by arm. We did not collect data on antibiotic usage outside of the study. Some children likely received antibiotics during the follow-up period, which could have affected results if antibiotic usage were differential by treatment group. If antibiotic usage was similar between arms, we would anticipate that results would be biased toward the null, which could mask some smaller effects of the azithromycin dose. We did not collect data on pneumococcal vaccine coverage in the study. Pneumococcal conjugate vaccine coverage is high in the study area and likely did not differ by treatment group. Swabs were only collected at baseline and 14 days and 6 months after treatment. Additional swab collection at different time points during the study may have helped to understand more precisely when selection for macrolide resistance begins to wane. Evaluation of resistance to other antibiotics not included in this study, such as cephalosporins or vancomycin, may have been useful to understand the full impact of azithromycin on antibiotic resistance patterns and could be considered in future study. This study evaluated a single oral dose of azithromycin dosed according to current treatment guidelines for trachoma and childhood mortality. Whether repeated dosing with azithromycin leads to a cumulative effect or more persistent selection for resistance cannot be evaluated in the current study.

A single oral dose of azithromycin selected for macrolide and β-lactam resistance in *S. pneumoniae* isolated from the nasopharynx of children in the short term, but not 6 months after treatment in children receiving azithromycin compared with placebo. Although surveillance of antimicrobial resistance following antibiotic treatment remains a high priority, the reduction of prevalence of resistance once selection pressure is removed is reassuring.
